# Antiphospholipid Antibody Syndrome in Childhood Systemic Lupus Erythematous With a Unique Presentation: A Case Report

**DOI:** 10.7759/cureus.27205

**Published:** 2022-07-24

**Authors:** Amit R Rup, Arun K Dash, Jyoti Ranjan Behera, Sanjay Kumar Sahu, Rama Krushna Gudu, Gummalla Gyandeep, Moparthi Puramjai, Prasanta Padhan, Mukesh K Jain

**Affiliations:** 1 Pediatrics, Kalinga Institute of Medical Sciences, Bhubaneswar, IND; 2 Department of Rheumatology, Kalinga Institute of Medical Sciences, Bhubaneswar, IND

**Keywords:** childhood, thrombosis, hemorrhage, mixing study, anti phospholipid syndrome, systemic lupus erythematosus

## Abstract

Initial presentation of childhood systemic lupus erythematosus (SLE) as antiphospholipid syndrome (APS) is uncommon; moreover, APS presenting with both hemorrhage and thrombosis is very rare.

We report a case of a previously healthy eight-year-old boy, without any significant past or family history, who presented with ecchymotic patches, epistaxis, and right-side hemiparesis. Investigation showed severe thrombocytopenia and isolated high activated partial thromboplastin time (aPTT) not corrected by mixing study. During his hospital stay, the child developed left-sided focal seizure and digital gangrene as thrombotic events.

Neuroimaging revealed initially hemorrhagic stroke and subsequently bilateral infarct of middle cerebral artery (MCA) territory. The child was diagnosed as a case of SLE with APS based on Systemic Lupus International Collaboration Clinics (SLICC) criteria, revised APS classification, clinicoimmunological profile and neuroimaging. As the child was progressing towards catastrophic APS, he was treated aggressively with intravenous pulse methylprednisolone, intravenous cyclophosphamide and plasmapheresis with successful recovery.

A simple bleeding manifestation may mask a serious disorder. A simple test like mixing study is helpful in diagnosis and in avoiding unnecessary investigations. A combination of both hemorrhage and thrombosis is an unusual presentation of APS and should always be suspected in case of autoimmune disorder, especially in SLE.

## Introduction

Antiphospholipid syndrome (APS) is a rare systemic autoimmune disease having an incidence of 2/1,00,000 population. It is characterized by thromboembolic phenomenon and pregnancy complications, secondary to connective tissue disorder, mainly systemic lupus erythematosus (SLE) [1]. According to 2006 criteria, APS with or without the presence of other risk factors for arterial and venous thrombosis is used in place of the previous classification of primary and secondary APS [2]. Catastrophic APS (CAPS) is a very severe form of APS seen in <1% cases, characterized by multiorgan failure due to massive small vessel thrombosis [3]. Bleeding is very unusual in APS, and if present, is mainly due to significant thrombocytopenia or disseminated intravascular coagulation.

## Case presentation

An eight-year-old boy, born to non-consanguineous parents, was admitted with complaints of bluish spots all over the body and nasal bleeding for seven days with weakness on the right side of the body for two days. There was no history of fever, jaundice, oliguria, seizure, trauma, blood transfusion, vaccination or recent drug intake. Two days prior to admission, there was a history of local bleeding following tooth extraction requiring one unit of blood transfusion. There was no significant past history or any bleeding disorder in the family. At the time of admission, the child was afebrile and hemodynamically stable. On general examination, the child had moderate pallor and generalized non-tender lymphadenopathy, without icterus, cyanosis, clubbing and edema. Epistaxis, gum bleeding and ecchymotic patches on extremities were noted.

On central nervous system (CNS) examination, higher function, cranial nerves, and sensory examination were normal. Hypotonia with power of 3/5 in both upper and lower limb, plantar extensor and exaggerated deep tendon reflexes were found on right side on motor examination. Rest of the CNS and other systemic examinations were normal. Based on the history and clinical findings of bleeding manifestations and right-sided incomplete hemiparesis, the differential diagnosis of immune thrombocytopenic purpura (ITP), or hematological malignancy was considered.

Management and outcome

**Figure 1:**
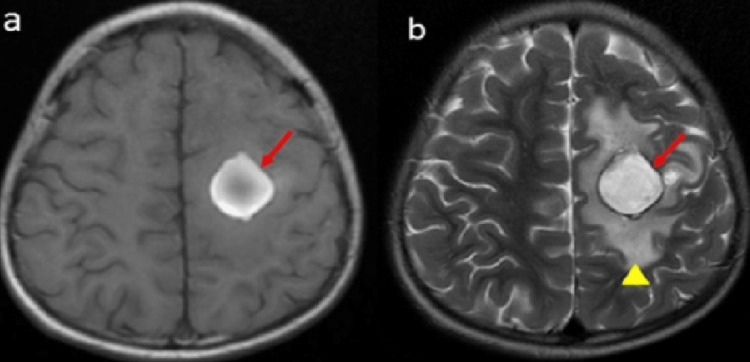
MRI showing T1 (a) and T2 (b) hyperintense well defined rounded lesion with surrounding edema in left centrum semiovale corresponding to left MCA territory suggestive of late sub-acute hemorrhage. MCA - middle cerebral artery

On investigation, total leukocyte counts were 11600/cumm (4000-11000/cumm), haemoglobin 9.5 gm/dl (11.5-14.5 gm/dl), platelet count 7000/cumm (N-150-400 ×103/cumm), activated partial thromboplastin time (aPTT) was 107 seconds (N 15-30 sec) with normal prothrombin time (PT). The urine routine revealed 4+ proteinuria and the spot urine protein creatinine ratio (PCR) was 27 (N < 0.2). Thrombocytopenia without any evidence of haemolysis was found on the peripheral smear. Other investigations like serum urea was 20 mg/dl, creatinine 0.5 mg/dl, bilirubin 1.5 mg/dl, serum glutamic-oxaloacetic transaminase (SGOT) 40 IU/L, serum glutamic-pyruvic transaminase (SGPT) 45 IU/L and serum procalcitonin was 0.2 mg/dl. A bone marrow study revealed no abnormality. Magnetic resonance imaging (MRI) of brain revealed left middle cerebral artery (MCA) territory sub-acute hemorrhage (Figure 1).

Considering isolated aPTT prolongation mixing study was performed, which showed partial correction of aPTT from 107 to 66 seconds (suggesting presence of an inhibitor of coagulation factor, rather than deficiency of coagulation factors). Isolated aPTT prolongation and normal bone marrow findings ruled out ITP and leukemia. Owing to partial correction of aPTT on mixing study, a test for the presence of antiphospholipid antibody (APLA) was done which showed the normal value of lupus anticoagulant and anti beta2 glycoprotein, but elevated anticardiolipin antibody IgG level 23.21 U/ml (Normal <10U/ml). The presence of significant proteinuria and raised anticardiolipin antibody led to suspicion of SLE. Further investigation revealed low C3 (39 mg/dl, normal 80-160mg/dl), positive anti-nuclear antibody (ANA) by immunofluorescence assay, and anti-double-stranded DNA antibody (431IU/ml, normal <100IU/ml) but direct coomb test was negative.

**Figure 2:**
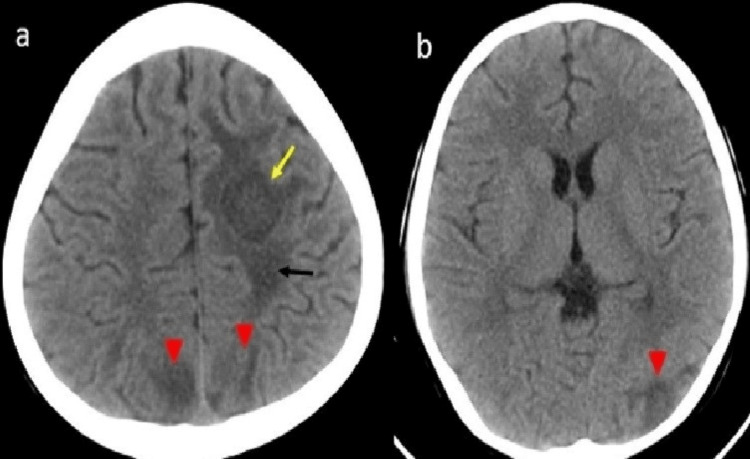
Non contrast CT Brain after new onset seizure showing (a) Iso to hypodense left centrum semiovale subacute hemorrhage with mild edema (b) Newly developed ill defined hypodensities involving cortex and sub-cortical regions in bilateral high parietal and left tempero-occipital lobe suggestive of acute infarcts.

Initially, the child was managed conservatively with intravenous fluids, packed red cells, and random donor platelets. On day five of admission, the child developed hypertension (blood pressure 120/88mmHg >95th percentile as per age, sex, height), digital gangrene, vasculitis rash, malar rash, and left focal seizure. CT scan of the brain showed bilateral MCA and left posterior cerebral artery (PCA) territory infarct (Figure 2). Based on these clinical findings and investigation a diagnosis of SLE with APS with lupus nephritis was done.

On day seven, the child was treated with pulse methylprednisolone @30mg/kg for three days for APS. Owing to progressive CNS worsening and development of digital gangrene, five cycles of plasmapheresis were done as a treatment protocol for probable CAPS. IV cyclophosphamide @ 500 mg/m2 monthly, and low molecular weight heparin (LMWH) were added in view of lupus nephritis and thrombotic events. With this treatment, the child developed significant clinical improvement with normal platelet counts of 2.9lakhs/cmm, and aPTT (26 seconds). However, proteinuria and hypertension persisted. The child was discharged with oral steroid 2mg/kg with hydroxyl chloroquine 5mg/kg, nifedipine, and warfarin. On three- and six-month follow-ups, complete blood count, PT/INR, aPTT, C3, ANA, anti-ds DNA antibody, and PCR were monitored. All parameters revealed improving trends. Lab parameters during hospitalization, discharge, and at three- and six-month follow-ups are depicted in Table 1.

**Table 1:** Serial investigations at diagnosis and follow up at 3 months & 6 months. Hb-Haemoglobin, TLC- Total leucocyte count, TPC-  Total platelet count, PT/INR- Prothrombin time/international normalized ratio, aPTT- Activated partial thromboplastin time, LFT- Liver function test, RFT- Renal function test, PCR- Urine protein creatinine ratio, FU- Follow up

Investigation	During Hospitalisation				At Diagnosis			Discharge	FU 1	FU 2
Hb (g/dl)	9.5	9.1	8.4	9.5	9.5	6.8	7.5	11.6	11.4	11.8
TLC(10^3^/Cumm)	11.6	8.5	8.27	7.9	14.4	8.5	7.2	20.3	16.6	13.9
TPC(10^3^/Cumm)	10	7	10	29	120	156	93	513	443	428
PT/INR	12/1.2	15/1.3	N	N	N		N	15/1.4	20/1.8	20/1.9
aPTT	101	107	115	105	58	-	26	28	24	29.4
PCR	27				26			10.35	6.75	3.78
dsDNA IU/ml					431			302		121
C3 mg/dl					39			69		76
Anti cardiolipin antibody IgG					23.2			13.2		11

Currently, the child is on oral prednisolone 20 mg/day, mycophenolate mofetil @600mg/m2, hydroxychloroquine @5 mg/kg, along with calcium supplementation.

## Discussion

APS is an acquired thrombophilic disorder characterized by recurrent venous/arterial thrombosis and/or pregnancy loss in the presence of APLA including lupus anticoagulant (LAC), and anticardiolipin (aCL) or anti-β2-glycoprotein-1 (anti-β2GP-1) [4]. APS may be primary or secondary to autoimmune diseases like SLE. In 38-87 % of patients with pediatric SLE, the antiphospholipid antibody has been reported, but APS is rare in children [5]. In one multicentric study cSLE-APS was observed in 67/1,519 (4%) and was diagnosed at disease onset in 39/67 (58%) [6]. Another case report by Carvalho et al. described CAPS as the initial presentation which evolves into childhood SLE [7].

According to the revised classification of APS, the index case had a stroke and digital gangrene (suggestive of vascular thrombosis) along with the presence of anticardiolipin IgG antibody in medium titer, hence diagnosed as APS [8]. Bleeding can be the first manifestation of APS but it is uncommon, and usually associated with thrombocytopenia [8]. The child’s initial presentation being hemorrhagic stroke followed by thrombotic complication (MCA territory infarction, left focal seizure, digital gangrene, hypertension with proteinuria) with positive anticardiolipin antibody qualifies for the definition of probable CAPS [2]. In one of the previously reported cases in the pediatric age group, the initial presentation was thrombotic microangiopathic hemolytic anemia (TMHA) associated with antiphospholipid antibodies who subsequently died due to CAPS [9]. But in the index case, the initial presentation was hemorrhagic stroke followed by thrombotic events, which was treated as a case of proven CAPS without waiting much for further progression of digital gangrene or organ failure.

CAPS is a rare variant of APS, occurring in less than 1% of cases, associated with multiple small vessel thrombosis resulting in multi-organ failure with high morbidity and mortality [10]. The validated diagnostic criteria of definite CAPS include: 1) Involvement of three or more organ systems, 2) Acute onset of less than one week 3) Histopathologic confirmation of small vessel occlusion in at least one organ/tissue, 4) Presence of APL. The striking feature of the syndrome is the presence of acute microangiopathy, rather than the large-vessel occlusions more typically observed in patients with both primary and secondary APS. CAPS can cause a systemic inflammatory response which leads to acute renal failure, acute respiratory distress syndrome (ARDS), cerebral edema, and myocardial dysfunction [11]. Early diagnosis is crucial since treatment with anticoagulants, steroids, plasma exchange (PE)/intravenous immunoglobulin and other immune modulators such as rituximab have been shown to improve outcomes [12]. Plasma exchange or intravenous immunoglobulin are used as a second-line treatment when the patient does not responds to first-line treatment like anticoagulant and steroids. Plasma exchange primarily acts by removal of pathogenic IgG aCL and β-2-glycoprotein-1, as well as cytokines, such as interleukin 1(IL-1), IL-6, tumor necrosis factor-α, and complement [13]. Since 2001, the increased use of anticoagulant + steroid + plasma exchange and/or IVIG has resulted in a 20% reduction in mortality, with a current mortality rate of 39% [14]. In a series of 230 cases of CAPS, treatment with PE was independently associated with decreased mortality (OR 0.36 [0.14-0.92] p = 0.033) [15]. Mortality in CAPS is high, but with early and effective treatment reduction has been noted recently [15]. Necessary investigations to be done during follow-up are complete blood count, C-reactive protein, ESR, C3 and C4 level, anti-ds DNA, serum creatinine, electrolytes such as sodium, potassium, calcium, spot urine PCR and blood sugar. Follow-up frequency is monthly for the first six months, then every three months up to two to three years. Ophthalmological evaluation is required every six to 12 months to look for retinal toxicity and color vision.

## Conclusions

This case had a unique presentation with both hemorrhage and thrombosis, which is rarely seen in pediatric SLE with APS. There was a rapid progression from APS to CAPS, which was treated aggressively with steroids, plasmapheresis, and cyclophosphamide resulting in a better outcome. In cases of deranged coagulation parameters, simple laboratory tests like a mixing study can provide important clues for the diagnosis of diseases like APS. Clinicians should suspect and screen for SLE when a diagnosis of APS is made and vice versa, which can prevent many life-threatening complications.

## References

[1] Mukherjee SS, Dhar S, Gaikward RP, Saha A (2015). Antiphospholipid antibody syndrome in pediatric population: an overview. Indian J Pediatr Dermatol.

[2] Miyakis S, Lockshin MD, Atsumi T (2006). International consensus statement on an update of the classification criteria for definite antiphospholipid syndrome (APS). J Thromb Haemost.

[3] Limper M, Scirè CA, Talarico R (2018). Antiphospholipid syndrome: state of the art on clinical practice guidelines. RMD Open.

[4] Ahluwalia J, Singh S, Naseem S (2014). Antiphospholipid antibodies in children with systemic lupus erythematosus: a long-term clinical and laboratory follow-up status study from northwest India. Rheumatol Int.

[5] Levine JS, Branch DW, Rauch J (2002). The antiphospholipid syndrome. N Engl J Med.

[6] Islabão AG, Mota LM, Ribeiro MC (2020). Childhood-onset systemic lupus erythematosus-related antiphospholipid syndrome: a multicenter study with 1519 patients. Autoimmun Rev.

[7] Carvalho JF, Silva FF, Shoenfeld Y (2021). Pediatric catastrophic antiphospholipid syndrome patient evolving to systemic lupus erythematosus. Lupus.

[8] Forastiero R (2012). Bleeding in the antiphospholipid syndrome. Hematology.

[9] Prasad N, Bhadauria D, Agarwal N, Gupta A, Gupta P, Jain M, Lal H (2012). Catastrophic antiphospholipid antibody syndrome in a child with thrombotic microangiopathy. Indian J Nephrol.

[10] Piette JC, Cervera R, Levy RA (2003). The catastrophic antiphospholipid syndrome- Asherson’s syndrome. Ann Med Interne (Paris).

[11] Erkan D, Cervera R, Asherson RA (2003). Catastrophic antiphospholipid syndrome: where do we stand?. Arthritis Rheum.

[12] Cervera R (2010). Catastrophic antiphospholipid syndrome (CAPS): update from the 'CAPS Registry'. Lupus.

[13] Cervera R, Asherson RA, Font J (2006). Catastrophic antiphospholipid syndrome. Rheum Dis Clin North Am.

[14] Bucciarelli S, Cervera R, Espinosa G, Gómez-Puerta JA, Ramos-Casals M, Font J (2006). Mortality in the catastrophic antiphospholipid syndrome: causes of death and prognostic factors. Autoimmun Rev.

[15] Westney GE, Harris EN (2002). Catastrophic antiphospholipid syndrome in the intensive care unit. Crit Care Clin.

